# (*E*)-3-[4-(Dimethyl­amino)­benzyl­idene]-2,3-di­hydro-1*H*,9*H*-pyrrolo­[2,1-*b*]quinazolin-9-one

**DOI:** 10.1107/S1600536810020878

**Published:** 2010-06-05

**Authors:** Burkhon Zh. Elmuradov, Rasul Ya. Okmanov, Asqar Sh. Abdurazakov, Bakhodir Tashkhodjaev, Khusnutdin M. Shakhidoyatov

**Affiliations:** a S. Yunusov Institute of the Chemistry of Plant Substances, Academy of Sciences of Uzbekistan, Mirzo Ulugbek Str. 77, Tashkent 100170, Uzbekistan

## Abstract

The title compound, C_20_H_19_N_3_O, was obtained by condensation of 2,3-dihydro-1*H*,9*H*-pyrrolo­[2,1-*b*]quinazolin-9-one (alkaloid de­oxy­vasicinone, isolated from *Peganum Harmala*) with 4-(dimethyl­amino)­benzaldehyde in the presence of sodium methoxide. The 2,3-dihydro-1*H*,9*H*-pyrrolo­[2,1-*b*]quinazolin-9-one part of the mol­ecule is roughly planar (r.m.s. deviation = 0.0178 Å) and is essentially coplanar with the benzil­idene ring (r.m.s. deviation = 0.0080 Å), forming a dihedral angle of 5.0 (1)°. The crystal structure is stabilized by two aromatic π–π stacking inter­actions observed between the benzene rings of neighboring mol­ecules [centroid–centroid distance = 3.7555 (19) Å.

## Related literature

For the synthesis of 2,3-dihydro-1*H*-pyrrolo­[2,1-*b*]quinazolin-9-one and the title compound, see: Shakhidoyatov *et al.* (1977 ▶); Elmuradov *et al.* (2009 ▶); Shakhidoyatov & Kaysarov, (1998 ▶); Jahng *et al.* (2008 ▶). For the physiological activity of 2,3-dihydro-1*H*-pyrrolo­[2,1-*b*]quinazolin-9-one and its derivatives, see: Chatterjee & Ganguly, (1968 ▶); Al-Shamma *et al.* (1981 ▶); Johne (1981 ▶); Telezhenetskaya & Yunusov, (1977 ▶); Yunusov *et al.* (1978 ▶). For related structures, see: Barnes *et al.* (1985 ▶); Wu *et al.* (1997 ▶).
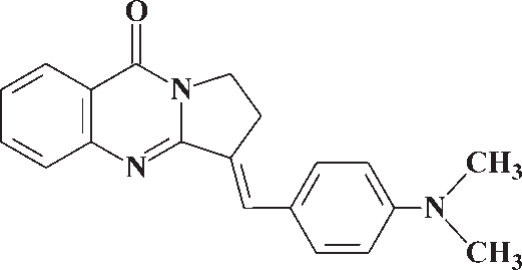


## Experimental

### 

#### Crystal data

C_20_H_19_N_3_O
*M* *_r_* = 317.38
Monoclinic, 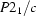

*a* = 8.8030 (18) Å
*b* = 16.415 (3) Å
*c* = 11.463 (2) Å
β = 105.05 (3)°
*V* = 1599.6 (6) Å^3^
*Z* = 4
Cu *K*α radiation
μ = 0.66 mm^−1^
*T* = 300 K
0.60 × 0.20 × 0.15 mm

#### Data collection

Stoe Stadi-4 four-circle diffractometer
Absorption correction: ψ scan (*X-RED*; Stoe & Cie, 1997 ▶). *T*_min_ = 0.854, *T*_max_ = 0.906
2446 measured reflections
2342 independent reflections
1728 reflections with *I* > 2σ(*I*)
θ_max_ = 60.0°
3 standard reflections every 60 min  intensity decay: 10.0%

#### Refinement

*R*[*F* ^2^ > 2σ(*F* ^2^)] = 0.057
*wR*(*F* ^2^) = 0.142
*S* = 1.13
2342 reflections
220 parameters
H-atom parameters constrained
Δρ_max_ = 0.16 e Å^−3^
Δρ_min_ = −0.18 e Å^−3^

### 

Data collection: *STADI4* (Stoe & Cie, 1997 ▶); cell refinement: *STADI4*; data reduction: *X-RED* (Stoe & Cie, 1997 ▶); program(s) used to solve structure: *SHELXS97* (Sheldrick, 2008 ▶); program(s) used to refine structure: *SHELXL97* (Sheldrick, 2008 ▶); molecular graphics: *XP* in *SHELXTL* (Sheldrick, 2008 ▶); software used to prepare material for publication: *SHELXL97* and *PLATON* (Spek, 2009 ▶).

## Supplementary Material

Crystal structure: contains datablocks I, global. DOI: 10.1107/S1600536810020878/si2264sup1.cif

Structure factors: contains datablocks I. DOI: 10.1107/S1600536810020878/si2264Isup2.hkl

Additional supplementary materials:  crystallographic information; 3D view; checkCIF report

## Figures and Tables

**Fig. 1.**
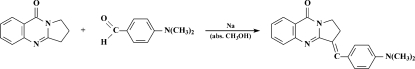
Reaction sequence.

**Fig. 2.**
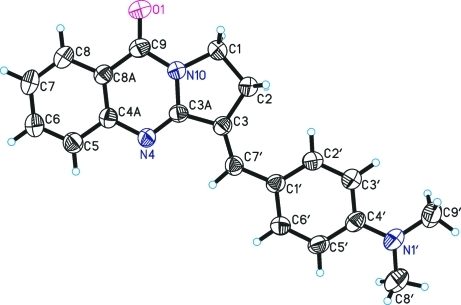
The molecular structure of the title compound, showing 50% probability displacement ellipsoids and the atom-numbering scheme.

**Fig. 3.**
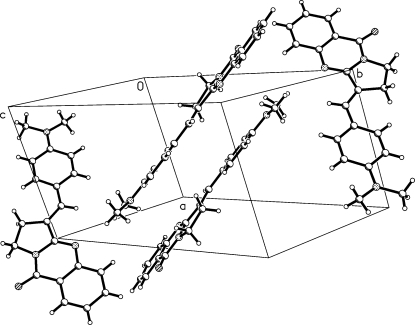
A section of molecular packing of the title compound.

## supplementary crystallographic information

### Comment

The derivatives of tricyclic quinazoline alkaloids possess different pharmacological activities (Chatterjee & Ganguly, 1968; Al-Shamma *et al.*, 1981; Johne, 1981; Telezhenetskaya & Yunusov, 1977; Yunusov *et al.*, 1978) and was found simples and convenient methods of a synthesis of these compounds (Shakhidoyatov *et al.*, 1977; Shakhidoyatov & Kaysarov, 1998; Jahng *et al.*, 2008).

Condensation of 2,3-dihydro-1*H*-pyrrolo[2,1-*b*]quinazolin-9-one (alkaloid Deoxyvasicinone, isolated from *Peganum Harmala*) (Chatterjee & Ganguly, 1968) with 4-dimethylaminobenzaldehyde at 278 (1) K in presence of sodium methoxide (Elmuradov *et al.*, 2009) leads to the formation of (*E*)-3-(4-dimethylamino)benzylidene-2,3-dihydro- 1*H*-pyrrolo[2,1-*b*]quinazolin-9-one (Figure 1).

The quinazoline part of molecule (C1/C2/C3/C3a/N4/C4a/C5/C6/C7/C8/C8a/C9/N10) is flat, r.m.s. deviation = 0.0178 Å, and benzilidene ring (C1'/C2'/C3'/C4'/C5'/C6'/C7') is also flat, r.m.s. deviation = 0.0080 Å, the angle between fragment planes is 5.0 (1)° (Figure 2). Torsion angle in fragment C3a–C3–C7'–C6' is 175.0 (5)° (Barnes *et al.*, 1985; Wu *et al.*, 1997), indicating the conjugation of π-electronic systems of pyrrolo(2,1-*b*)quinazolone and benzilidene rings. The sum of valent angles of all nitrogen atoms are close to 360° (Figure 3) which specifies *sp*^2^ hybridizations of nitrogen atoms. It specifies, that the lone electronic pair of nitrogen atoms participate in a conjugation with π electrons of aromatic ring. The crystal structure is stabilized by *π*-*π* interactions between the benzene rings of neighbor standing molecules with the distance *Cg*3···*Cg*4^i^ = 3.7555 (19) Å [symmetry code: (i) -*x*, 1 - *y*, 1 - *z*; *Cg*3 and *Cg*4 are centroid of the C1'-C6' and C4a/C5-C8/C8a two benzene rings].

### Experimental

0.115 g sodium (5 mmole) was dissolved in 5 ml absolute methanol, and 0.186 g (1 mmole) of 2,3-dihydro-1*H*-pyrrolo[2,1-*b*]quinazolin-9-one and 0.151 g (1 mmole) 4-dimethylamino-benzaldehyde were added (Figure 1). Reaction mixture was left at 278 (1) K for 3 weeks. The dropped out single crystals, suitable for X-ray analysis were filtered, flushed at first with alcohol, then water. 0.05 g (16%) of the title compound was obtained in the reaction, m.p. 514-516 K.

### Refinement

The 10% decay correction was applied by using the programm *X-RED*. The H atoms bonded to C atoms were placed geometrically (with C–H distances of 0.98 Å for CH; 0.97 Å for CH_2_; 0.96 Å for CH_3_; and 0.93 Å for C_ar_) and included in the refinement in a riding motion approximation with *U*_iso_(H)=1.2*U*_eq_(C) [*U*_iso_(H)=1.5*U*_eq_(C) for methyl H atoms].

### Crystal data

**Table:**

C_20_H_19_N_3_O	*F*(000) = 672
*M**_r_* = 317.38	*D*_x_ = 1.318 Mg m^−^^3^
Monoclinic, *P*2_1_/*c*	Melting point: 514(2) K
Hall symbol: -P 2ybc	Cu *K*α radiation, λ = 1.54184 Å
*a* = 8.8030 (18) Å	Cell parameters from 12 reflections
*b* = 16.415 (3) Å	θ = 10–20°
*c* = 11.463 (2) Å	µ = 0.66 mm^−^^1^
β = 105.05 (3)°	*T* = 300 K
*V* = 1599.6 (6) Å^3^	Prizmatic, light yellow
*Z* = 4	0.60 × 0.20 × 0.15 mm

### Data collection

**Table:**

Stoe Stadi-4 four-circle diffractometer	1728 reflections with *I* > 2σ(*I*)
Radiation source: fine-focus sealed tube	*R*_int_ = 0.0000
graphite	θ_max_ = 60.0°, θ_min_ = 4.8°
Scan width (ω) = 1.32 – 1.56, scan ratio 2θ:ω = 0.00 I(Net) and sigma(I) calculated according to Blessing (1987)	*h* = −9→9
Absorption correction: ψ scan (*X-RED*; Stoe & Cie, 1997).	*k* = 0→18
*T*_min_ = 0.854, *T*_max_ = 0.906	*l* = 0→12
2446 measured reflections	3 standard reflections every 60 min
2342 independent reflections	intensity decay: 10.0%

### Refinement

**Table:**

Refinement on *F*^2^	Secondary atom site location: difference Fourier map
Least-squares matrix: full	Hydrogen site location: inferred from neighbouring sites
*R*[*F*^2^ > 2σ(*F*^2^)] = 0.057	H-atom parameters constrained
*wR*(*F*^2^) = 0.142	*w* = 1/[σ^2^(*F*_o_^2^) + (0.0446*P*)^2^ + 1.0639*P*] where *P* = (*F*_o_^2^ + 2*F*_c_^2^)/3
*S* = 1.13	(Δ/σ)_max_ < 0.001
2342 reflections	Δρ_max_ = 0.16 e Å^−^^3^
220 parameters	Δρ_min_ = −0.18 e Å^−^^3^
0 restraints	Extinction correction: *SHELXL97* (Sheldrick, 2008), Fc^*^=kFc[1+0.001xFc^2^λ^3^/sin(2θ)]^-1/4^
Primary atom site location: structure-invariant direct methods	Extinction coefficient: 0.0033 (3)

### Special details

**Table:**

Experimental. Empirical absorption correction using ψ Scan. Reflections used Mu * R = 0.00 H K L, θ, χ, I~min~/I~max~: -2 1 1 21.1 80.5 0.900
Geometry. All e.s.d.'s (except the e.s.d. in the dihedral angle between two l.s. planes) are estimated using the full covariance matrix. The cell e.s.d.'s are taken into account individually in the estimation of e.s.d.'s in distances, angles and torsion angles; correlations between e.s.d.'s in cell parameters are only used when they are defined by crystal symmetry. An approximate (isotropic) treatment of cell e.s.d.'s is used for estimating e.s.d.'s involving l.s. planes.
Refinement. Refinement of *F*^2^ against ALL reflections. The weighted *R*-factor *wR* and goodness of fit *S* are based on *F*^2^, conventional *R*-factors *R* are based on *F*, with *F* set to zero for negative *F*^2^. The threshold expression of *F*^2^ > σ(*F*^2^) is used only for calculating *R*-factors(gt) *etc*. and is not relevant to the choice of reflections for refinement. *R*-factors based on *F*^2^ are statistically about twice as large as those based on *F*, and *R*- factors based on ALL data will be even larger.

### Fractional atomic coordinates and isotropic or equivalent isotropic displacement parameters (Å^2^)

**Table:**

	*x*	*y*	*z*	*U*_iso_*/*U*_eq_	
O1	−0.1964 (3)	0.57316 (13)	0.07780 (19)	0.0553 (7)	
C1	0.0365 (4)	0.48250 (18)	0.2373 (3)	0.0496 (9)	
H1A	0.0818	0.4880	0.1691	0.060*	
H1B	−0.0432	0.4402	0.2197	0.060*	
C2	0.1634 (4)	0.46318 (18)	0.3530 (3)	0.0435 (8)	
H2A	0.1435	0.4107	0.3851	0.052*	
H2B	0.2663	0.4617	0.3370	0.052*	
C3	0.1562 (3)	0.52982 (17)	0.4410 (3)	0.0373 (7)	
C3A	0.0343 (3)	0.58741 (17)	0.3798 (2)	0.0355 (7)	
N4	−0.0078 (3)	0.65349 (14)	0.4246 (2)	0.0391 (6)	
C4A	−0.1278 (3)	0.69758 (17)	0.3470 (3)	0.0378 (7)	
C5	−0.1799 (4)	0.76888 (18)	0.3914 (3)	0.0459 (8)	
H5A	−0.1330	0.7856	0.4700	0.055*	
C6	−0.2995 (4)	0.8144 (2)	0.3201 (3)	0.0495 (8)	
H6A	−0.3327	0.8618	0.3503	0.059*	
C7	−0.3707 (4)	0.78958 (19)	0.2026 (3)	0.0476 (8)	
H7A	−0.4525	0.8201	0.1549	0.057*	
C8	−0.3213 (3)	0.72058 (19)	0.1568 (3)	0.0452 (8)	
H8A	−0.3690	0.7047	0.0780	0.054*	
C8A	−0.1995 (3)	0.67373 (17)	0.2279 (3)	0.0379 (7)	
C9	−0.1474 (3)	0.59996 (18)	0.1804 (3)	0.0413 (7)	
N10	−0.0298 (3)	0.55973 (14)	0.2639 (2)	0.0384 (6)	
N1'	0.7209 (3)	0.34893 (18)	0.8577 (2)	0.0585 (8)	
C1'	0.3643 (3)	0.49063 (17)	0.6292 (2)	0.0375 (7)	
C2'	0.4284 (4)	0.42189 (18)	0.5885 (3)	0.0445 (8)	
H2'A	0.3902	0.4064	0.5081	0.053*	
C3'	0.5455 (4)	0.37605 (19)	0.6621 (3)	0.0451 (8)	
H3'A	0.5847	0.3310	0.6303	0.054*	
C4'	0.6065 (3)	0.39601 (18)	0.7836 (3)	0.0426 (8)	
C5'	0.5453 (4)	0.46547 (19)	0.8262 (3)	0.0462 (8)	
H5'A	0.5839	0.4812	0.9064	0.055*	
C6'	0.4282 (3)	0.51068 (18)	0.7500 (3)	0.0423 (8)	
H6'A	0.3904	0.5566	0.7808	0.051*	
C7'	0.2395 (3)	0.54038 (17)	0.5554 (3)	0.0383 (7)	
H7'A	0.2135	0.5864	0.5934	0.046*	
C8'	0.7668 (5)	0.3632 (3)	0.9855 (3)	0.0906 (15)	
H8'A	0.6753	0.3623	1.0164	0.136*	
H8'B	0.8171	0.4154	1.0015	0.136*	
H8'C	0.8386	0.3214	1.0243	0.136*	
C9'	0.7788 (4)	0.2766 (2)	0.8122 (3)	0.0617 (10)	
H9'A	0.8256	0.2912	0.7482	0.093*	
H9'B	0.6932	0.2397	0.7818	0.093*	
H9'C	0.8562	0.2509	0.8762	0.093*	

### Atomic displacement parameters (Å^2^)

**Table:**

	*U*^11^	*U*^22^	*U*^33^	*U*^12^	*U*^13^	*U*^23^
O1	0.0632 (15)	0.0588 (14)	0.0371 (13)	0.0057 (12)	0.0006 (11)	−0.0039 (11)
C1	0.056 (2)	0.0412 (18)	0.0468 (19)	0.0081 (16)	0.0043 (16)	−0.0075 (15)
C2	0.0460 (18)	0.0406 (18)	0.0415 (17)	0.0033 (15)	0.0072 (15)	−0.0009 (14)
C3	0.0345 (16)	0.0388 (16)	0.0379 (16)	−0.0011 (13)	0.0079 (13)	0.0008 (13)
C3A	0.0346 (16)	0.0360 (16)	0.0353 (15)	−0.0039 (13)	0.0079 (13)	−0.0016 (13)
N4	0.0348 (14)	0.0373 (14)	0.0418 (14)	0.0027 (11)	0.0039 (11)	−0.0016 (11)
C4A	0.0363 (17)	0.0348 (16)	0.0427 (17)	−0.0020 (13)	0.0111 (14)	0.0033 (13)
C5	0.0415 (18)	0.0440 (19)	0.0507 (19)	0.0007 (15)	0.0093 (15)	−0.0026 (15)
C6	0.0465 (19)	0.0432 (18)	0.059 (2)	0.0066 (15)	0.0149 (17)	0.0039 (16)
C7	0.0395 (18)	0.0464 (19)	0.057 (2)	0.0056 (15)	0.0124 (15)	0.0178 (16)
C8	0.0378 (17)	0.0506 (19)	0.0455 (18)	0.0015 (15)	0.0079 (14)	0.0109 (15)
C8A	0.0375 (17)	0.0371 (17)	0.0384 (16)	−0.0026 (13)	0.0088 (13)	0.0044 (13)
C9	0.0400 (18)	0.0453 (18)	0.0370 (17)	−0.0056 (14)	0.0072 (14)	0.0017 (14)
N10	0.0401 (14)	0.0368 (14)	0.0356 (13)	0.0023 (11)	0.0051 (11)	−0.0017 (11)
N1'	0.0514 (17)	0.0607 (19)	0.0523 (17)	0.0112 (15)	−0.0062 (14)	0.0008 (14)
C1'	0.0340 (16)	0.0379 (17)	0.0383 (16)	−0.0034 (13)	0.0052 (13)	−0.0011 (13)
C2'	0.0471 (19)	0.0445 (18)	0.0385 (17)	−0.0001 (15)	0.0054 (14)	−0.0029 (14)
C3'	0.0454 (19)	0.0449 (18)	0.0431 (18)	0.0035 (15)	0.0082 (15)	0.0014 (15)
C4'	0.0336 (17)	0.0441 (18)	0.0462 (18)	−0.0052 (14)	0.0033 (14)	0.0058 (15)
C5'	0.0423 (18)	0.0491 (19)	0.0414 (18)	−0.0042 (16)	0.0004 (15)	−0.0028 (15)
C6'	0.0411 (17)	0.0412 (17)	0.0426 (17)	−0.0020 (14)	0.0071 (14)	−0.0046 (14)
C7'	0.0347 (16)	0.0368 (16)	0.0430 (17)	0.0010 (13)	0.0096 (14)	−0.0007 (13)
C8'	0.092 (3)	0.103 (3)	0.056 (2)	0.034 (3)	−0.017 (2)	0.000 (2)
C9'	0.048 (2)	0.055 (2)	0.075 (2)	0.0088 (18)	0.0019 (18)	0.0083 (19)

### Geometric parameters (Å, °)

**Table:**

O1—C9	1.225 (3)	C8A—C9	1.450 (4)
C1—N10	1.461 (4)	C9—N10	1.382 (4)
C1—C2	1.529 (4)	N1'—C4'	1.375 (4)
C1—H1A	0.9700	N1'—C8'	1.434 (4)
C1—H1B	0.9700	N1'—C9'	1.442 (4)
C2—C3	1.500 (4)	C1'—C6'	1.392 (4)
C2—H2A	0.9700	C1'—C2'	1.395 (4)
C2—H2B	0.9700	C1'—C7'	1.452 (4)
C3—C7'	1.338 (4)	C2'—C3'	1.374 (4)
C3—C3A	1.465 (4)	C2'—H2'A	0.9300
C3A—N4	1.295 (3)	C3'—C4'	1.395 (4)
C3A—N10	1.378 (3)	C3'—H3'A	0.9300
N4—C4A	1.394 (4)	C4'—C5'	1.402 (4)
C4A—C5	1.400 (4)	C5'—C6'	1.381 (4)
C4A—C8A	1.403 (4)	C5'—H5'A	0.9300
C5—C6	1.374 (4)	C6'—H6'A	0.9300
C5—H5A	0.9300	C7'—H7'A	0.9300
C6—C7	1.390 (4)	C8'—H8'A	0.9600
C6—H6A	0.9300	C8'—H8'B	0.9600
C7—C8	1.366 (4)	C8'—H8'C	0.9600
C7—H7A	0.9300	C9'—H9'A	0.9600
C8—C8A	1.397 (4)	C9'—H9'B	0.9600
C8—H8A	0.9300	C9'—H9'C	0.9600
			
N10—C1—C2	103.9 (2)	C3A—N10—C9	123.8 (2)
N10—C1—H1A	111.0	C3A—N10—C1	113.7 (2)
C2—C1—H1A	111.0	C9—N10—C1	122.5 (2)
N10—C1—H1B	111.0	C4'—N1'—C8'	120.5 (3)
C2—C1—H1B	111.0	C4'—N1'—C9'	120.7 (3)
H1A—C1—H1B	109.0	C8'—N1'—C9'	118.2 (3)
C3—C2—C1	106.4 (2)	C6'—C1'—C2'	115.5 (3)
C3—C2—H2A	110.4	C6'—C1'—C7'	119.7 (3)
C1—C2—H2A	110.4	C2'—C1'—C7'	124.8 (3)
C3—C2—H2B	110.4	C3'—C2'—C1'	122.8 (3)
C1—C2—H2B	110.4	C3'—C2'—H2'A	118.6
H2A—C2—H2B	108.6	C1'—C2'—H2'A	118.6
C7'—C3—C3A	122.2 (3)	C2'—C3'—C4'	121.1 (3)
C7'—C3—C2	130.2 (3)	C2'—C3'—H3'A	119.4
C3A—C3—C2	107.6 (2)	C4'—C3'—H3'A	119.4
N4—C3A—N10	124.8 (3)	N1'—C4'—C3'	121.0 (3)
N4—C3A—C3	126.9 (3)	N1'—C4'—C5'	121.8 (3)
N10—C3A—C3	108.3 (2)	C3'—C4'—C5'	117.1 (3)
C3A—N4—C4A	115.4 (2)	C6'—C5'—C4'	120.5 (3)
N4—C4A—C5	117.9 (3)	C6'—C5'—H5'A	119.8
N4—C4A—C8A	123.4 (3)	C4'—C5'—H5'A	119.8
C5—C4A—C8A	118.7 (3)	C5'—C6'—C1'	123.0 (3)
C6—C5—C4A	120.7 (3)	C5'—C6'—H6'A	118.5
C6—C5—H5A	119.6	C1'—C6'—H6'A	118.5
C4A—C5—H5A	119.6	C3—C7'—C1'	129.6 (3)
C5—C6—C7	120.0 (3)	C3—C7'—H7'A	115.2
C5—C6—H6A	120.0	C1'—C7'—H7'A	115.2
C7—C6—H6A	120.0	N1'—C8'—H8'A	109.5
C8—C7—C6	120.4 (3)	N1'—C8'—H8'B	109.5
C8—C7—H7A	119.8	H8'A—C8'—H8'B	109.5
C6—C7—H7A	119.8	N1'—C8'—H8'C	109.5
C7—C8—C8A	120.3 (3)	H8'A—C8'—H8'C	109.5
C7—C8—H8A	119.8	H8'B—C8'—H8'C	109.5
C8A—C8—H8A	119.8	N1'—C9'—H9'A	109.5
C8—C8A—C4A	119.8 (3)	N1'—C9'—H9'B	109.5
C8—C8A—C9	120.7 (3)	H9'A—C9'—H9'B	109.5
C4A—C8A—C9	119.5 (3)	N1'—C9'—H9'C	109.5
O1—C9—N10	120.5 (3)	H9'A—C9'—H9'C	109.5
O1—C9—C8A	126.4 (3)	H9'B—C9'—H9'C	109.5
N10—C9—C8A	113.1 (3)		
